# Synthesis and biologic properties of hydrophilic sapphyrins, a new class of tumor-selective inhibitors of gene expression

**DOI:** 10.1186/1476-4598-6-9

**Published:** 2007-01-19

**Authors:** Zhong Wang, Philip S Lecane, Patricia Thiemann, Qing Fan, Cecilia Cortez, Xuan Ma, Danielle Tonev, Dale Miles, Louie Naumovski, Richard A Miller, Darren Magda, Dong-Gyu Cho, Jonathan L Sessler, Brian L Pike, Samantha M Yeligar, Mazen W Karaman, Joseph G Hacia

**Affiliations:** 1 Pharmacyclics, Inc., Sunnyvale, California, USA; 2 Department of Chemistry and Biochemistry, University of Texas at Austin, Austin, Texas, USA; 3 Department of Biochemistry and Molecular Biology, University of Southern California, Los Angeles, California, USA

## Abstract

**Background:**

Sapphyrin analogues and related porphyrin-like species have attracted attention as anticancer agents due to their selective localization in various cancers, including hematologic malignancies, relative to surrounding tissues. Sapphyrins are electron affinic compounds that generate high yields of singlet oxygen formation. Although initially explored in the context of photodynamic therapy, sapphyrins have intrinsic anticancer activity that is independent of their photosensitizing properties. However, the mechanisms for their anticancer activity have not been fully elucidated.

**Results:**

We have prepared a series of hydrophilic sapphyrins and evaluated their effect on proliferation, uptake, and cell death in adherent human lung (A549) and prostate (PC3) cancer cell lines and in an A549 xenograft tumor model. PCI-2050, the sapphyrin derivative with the highest *in vitro *growth inhibitory activity, significantly lowered 5-bromo-2'-deoxyuridine incorporation in S-phase A549 cells by 60% within eight hours and increased levels of reactive oxygen species within four hours. The growth inhibition pattern of PCI-2050 in the National Cancer Institute 60 cell line screen correlated most closely using the COMPARE algorithm with known transcriptional or translational inhibitors. Gene expression analyses conducted on A549 plateau phase cultures treated with PCI-2050 uncovered wide-spread decreases in mRNA levels, which especially affected short-lived transcripts. Intriguingly, PCI-2050 increased the levels of transcripts involved in RNA processing and trafficking, transcriptional regulation, and chromatin remodeling. We propose that these changes reflect the activation of cellular processes aimed at countering the observed wide-spread reductions in transcript levels. In our A549 xenograft model, the two lead compounds, PCI-2050 and PCI-2022, showed similar tumor distributions despite differences in plasma and kidney level profiles. This provides a possible explanation for the better tolerance of PCI-2022 relative to PCI-2050.

**Conclusion:**

Hydrophilic sapphyrins were found to display promise as novel agents that localize to tumors, generate oxidative stress, and inhibit gene expression.

## Background

Sapphyrins are a class of expanded porphyrins that were first discovered as an unanticipated product during the synthesis of vitamin B12 [1]. Subsequently, efficient chemical synthesis of these compounds [2,3] led to the discovery that sapphyrins can function as highly effective anion binding receptors [4]. Sapphyrins possess a 22 π-electron aromatic conjugation pathway and as a consequence are more electron affinic than the corresponding 18 π-electron porphyrin systems [5]. Due to a relatively high yield of singlet oxygen formation and near infrared absorbance properties, their potential as therapeutic agents was initially explored in the context of photodynamic therapy [6,7]. In the course of these studies, it was determined that sapphyrins can selectively accumulate in tumors, relative to surrounding tissues, similar to porphyrins and other expanded porphyrin systems such as the texaphyrins [8]. It was also noted that sapphyrins could display a significant degree of inhibitory activity in cellular assays even in the absence of light [9]. Recently, we reported the anti-cancer activity of several sapphyrin derivatives in hematologic cell lines and tumors, confirming that sapphyrins possess intrinsic anticancer activity that is independent of their photosensitizing properties [10,11].

In this report, we describe the preparation of a series of hydrophilic sapphyrin derivatives and the results of their evaluation as anti-cancer agents in adherent cell lines and a xenograft tumor model. We find that sapphyrin treatment leads to an increase in intracellular reactive oxygen species (ROS) and inhibits cell proliferation at early times, leading to cell death upon more prolonged treatment. Moreover, we have characterized gene expression profiles of human lung cancer cells cultured in the presence of the most active derivative in order to improve our understanding of the mechanism of action of these compounds. Sapphyrin-treated cells showed a wide-spread inhibition of gene expression, with short lived mRNAs displaying the most prominent decreases. These data lead us to suggest that sapphyrins act as global inhibitors of gene expression and that this plays a major role in their anti-cancer activity.

## Results

### Chemical synthesis of hydrophilic sapphyrins

**Figure 1:**
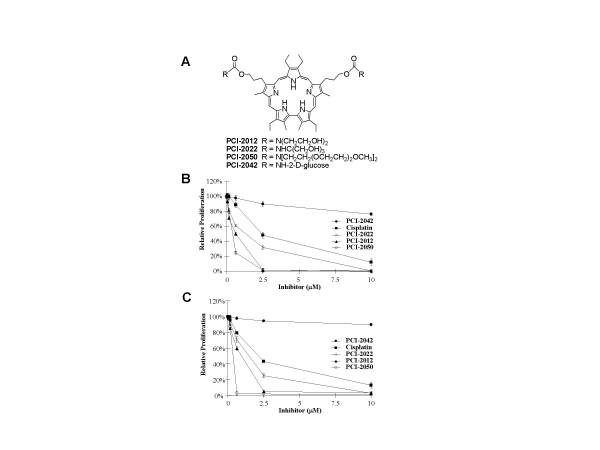
Effect of sapphyrins on cell proliferation. *A*, Structure of sapphyrins. *B*, A549 and *C*, PC3 cancer cells were treated with inhibitors for 24 hours, medium was replaced, and relative proliferation measured after an additional 48 hours by tetrazolium salt reduction. The anti-proliferative activity of cisplatin is shown for comparison.

Hydrophilic sapphyrin derivatives were prepared by condensation of a dihydroxylated sapphyrin precursor [3] with the corresponding tri(ethyleneglycol)methyl ether (PEG) or hydroxyl substituted amines using an active carbonate method (Fig. 1A and Additional File 1). The purity of all products was >90% as determined by reversed phase HPLC analysis. Sapphyrins were formulated for biological studies in 5% aqueous mannitol and quantified in methanol using the extinction coefficient determined for the parent compound (^443 ^ε = 598,782 M^-1 ^cm^-1^).

### Antiproliferative activity of hydrophilic sapphyrins

**Figure 2:**
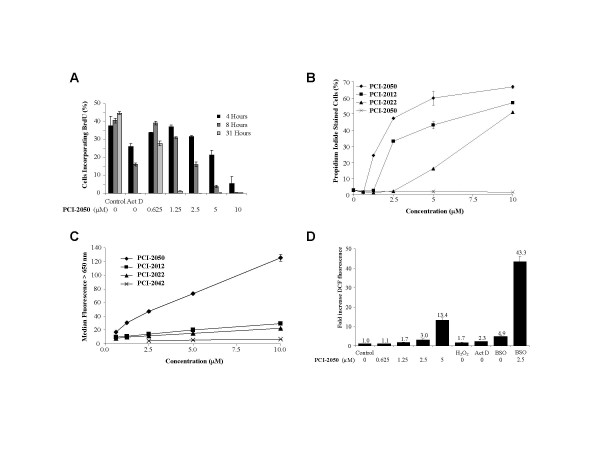
Biological effects of sapphyrins on A549 lung cancer cells. *A*, 5-Bromo-2'-deoxyuridine (BrdU) incorporation after exposure to PCI-2050 at indicated concentrations. The effect of actinomycin D (ActD, 200 ng/mL) treatment is shown for comparison. *B*, Cells were treated with inhibitors for 24 hours, medium was replaced, and viability measured after an additional 48 hours by propidium iodide staining. *C*, Cells were treated with sapphyrins for 4 hours, washed, and sapphyrin uptake estimated by cellular fluorescence above 650 nm. *D*, Cells were treated with PCI-2050 at indicated concentrations for 4 hours, washed, treated with dichlorofluorescin diacetate, and dichlorofluorescein fluorescence (DCF) measured in live-gated cells. Effect of hydrogen peroxide (H_2_O_2_, 200 μM), actinomycin D (Act D, 5 μg/mL), and pretreatment with L-buthionein- [S,R]-sulfoximine (BSO, 100 μM) for 24 hours are shown for comparison.

The hydrophilic sapphyrins considered in this study all displayed antiproliferative activity in A549 cells with a 50% growth inhibitory concentration (GI_50_) between ca. 0.5 and 2.5 μM measured 3 days after a 24 hour treatment with the exception of PCI-2042 which was essentially inactive (Fig. 1B). The order of compound activity was PCI-2050 > PCI-2012 > PCI-2022 > PCI-2042. Similar activity was observed in PC3 cells (Fig. 1C). Thus, antiproliferative activity in cultured cells correlated inversely with the number of hydroxyl group substituents. Treatment with 2.5 μM sapphyrin PCI-2050 reduced BrdU incorporation in S-phase A549 cells by 60% within 8 hours (Fig. 2A).

### Sapphyrin cell uptake and cytotoxicity

Cytotoxicity and uptake properties were examined in this set of related sapphyrins (Fig. 2B–C). Treatment of A549 cells with 1.25 μM or 2.5 μM sapphyrin PCI-2050 led to 25% or 50% cell death, respectively, within 3 days as measured using propidium iodide staining. A comparable degree of cell death (40%) was obtained with 200 ng/mL (0.16 μM) actinomycin D (data not shown). Other sapphyrins tested (PCI-2012, PCI-2022, and PCI-2042) caused less cell death, with activity that correlated inversely with the number of hydroxyl group substituents. Uptake of sapphyrins into cells was estimated based on their fluorescence in the near-infrared (>650 nm) channel of the flow cytometer (Fig. 2C) [11]. Cell-associated sapphyrin fluorescence after four hours of exposure to the four compounds correlated well with levels of cell death after 72 hours. These data lead us to suggest that the antiproliferative and cytotoxic activities of sapphyrin congeners are largely determined by the rate of their cellular uptake.

### Formation of Reactive Oxygen Species (ROS) in A549 cells

The most active derivative, sapphyrin PCI-2050, was selected for further mechanistic studies in vitro. A549 cells treated with sapphyrin PCI-2050 displayed a dose-dependent increase of intracellular peroxides (ROS) as measured by the conversion of dichlorofluorescin acetate (DCFA) to dichlorofluorescein (DCF) after four hours of treatment (Fig. 2D) [12]. DCF signal following treatment with 1.25 μM sapphyrin PCI-2050 was comparable to that following treatment with 200 μM hydrogen peroxide. To assess the involvement of the glutathione-dependent cellular antioxidant system, control cells were treated with L-buthionein- [S,R]-sulfoximine (BSO) overnight prior to sapphyrin treatment. BSO-treated cultures displayed a five-fold increase in DCF signal as compared to background, whereas cultures treated with 2.5 μM sapphyrin PCI-2050 and BSO showed a 43-fold increase as compared to the threefold increase seen upon exposure to 2.5 μM of sapphyrin PCI-2050 in the absence of BSO. This observation is interpreted in terms of treatment with sapphyrin PCI-2050 leading to ROS formation within 4 hours, an effect that is partially masked by glutathione-dependent pathways. Treatment with 5 μg/mL actinomycin D also led to ROS formation, although to a lesser degree than sapphyrin PCI-2050 at similar (5 μM) concentration.

### National cancer institute cell line screen

Testing of PCI-2050 in the NCI Developmental Therapeutics Program tumor cell line panel revealed broad overall activity with an overall Log 50% growth inhibition (Log GI_50_) of -7.04 with a range of 2.34 (Additional File 1). The COMPARE algorithm [13] was used to identify compounds present in the NCI synthetic compound database with GI_50 _values correlating most closely to those of PCI-2050. The most similar compounds (NCI accession number, Pearson correlation coefficient) obtained from this analysis were deoxybouvardin (NSC 259969, 0.841), bouvardin (NSC 259968, 0.809), S-3'-deacetyl-phyllanthoside (NSC 342443, 0.807), and olivomycin (NSC 38270, 0.797). Interestingly, these compounds all have been shown to induce global inhibition of transcription or translation in eukaryotic cells [14-16].

### Gene expression in sapphyrin-treated A549 cells

Plateau phase A549 cells treated with 2.5 μM PCI-2050 for four hours showed a global inhibition of gene expression with 4.7-fold more transcripts significantly down-regulated than were up-regulated in response to drug (801 versus 170). Experiments conducted with a lower (1.25 μM) dosage of PCI-2050 provided similar results with a 1.9-fold excess of down-regulated relative to up-regulated transcripts being seen (2,221 versus 1,187). Nevertheless, as can be inferred from the difference seen at 2.5 and 1.25 μM, the relative proportion of down-regulated transcripts is sensitive to drug dosage. Interestingly, reportedly short-lived transcripts (e.g. *MYC*, *ZNF217*, and *PNRC1*) [17] displayed prominent decreases (>3.5-fold, Benjamini-Hochberg corrected p < 0.01) in their levels in response to both dosages of PCI-2050. Conversely, according to our criteria, the levels of reportedly longer lived transcripts [17], such as *ATP6V1A*, *GNS*, and *EIF2S3*, were not significantly affected by either of these treatments.

Based on the these observations, one would predict that the expression profiles of plateau phase A549 cells treated with appropriate dosages of PCI-2050 or the transcription-inhibiting agent actinomycin D would be similar. Here, treatment with 5 μg/ml actinomycin D resulted in a 23.0-fold enrichment for down-regulated relative to up-regulated transcripts (2,281 versus 99, respectively). Interestingly, 42.8% of the 971 transcripts significantly changed by 2.5 μM PCI-2050 were also changed in the same direction by actinomycin D (Additional File 2). Conversely, 17.5% of the 2,380 transcripts significantly changed by actinomycin D were changed in the same direction by 2.5 μM PCI-2050 (Additional File 2).

**Figure 3:**
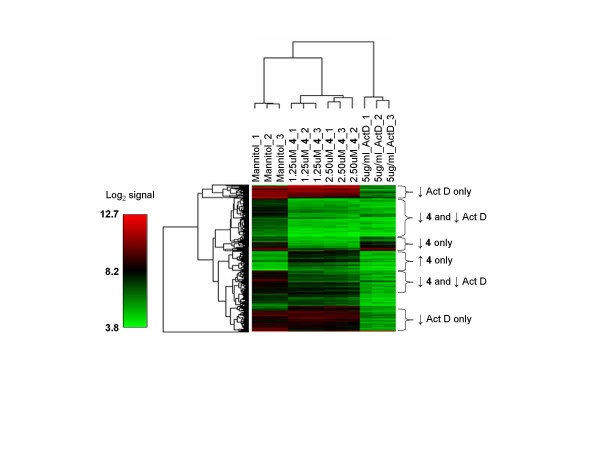
Hierarchical clustering analysis of gene expression data from drug-treated A549 cell cultures. The dendrograms were generated based on average linkage hierarchical clustering of expression data from 422 probe tilings whose coefficient of variation was greater than 0.15 across all groups. Data from untreated (mannitol) and drug-treated [(sapphyrin PCI-2050 = 2050) or (actinomycin D = ActD)] A549 cultures are provided in triplicate using the noted concentrations. The relative expression levels (↑ = up-regulated, ↓ = down-regulated) of selected gene groupings in drug-treated versus control cells are provided on the right side of the figure. Note that this reflects relationships only within the subset of genes used for cluster analysis.

Although the gene expression profiles of PCI-2050 and actinomycin D treated cells both reflect wide-spread decreases in mRNA levels, there were intriguing differences, as highlighted by hierarchical clustering analysis (Fig. 3). For example, the expression profiles of PCI-2050 treated samples were more closely related to those of the untreated controls than to actinomycin D treated samples. These sample groupings were driven by the behavior of distinct subsets of genes. For example, 94 transcripts were up-regulated in response to 1.25 μM PCI-2050 but down-regulated in response to 5 μg/ml actinomycin D (Additional File 3). In fact, 25 transcripts were down-regulated by actinomycin D but up-regulated by both dosages of PCI-2050 (Additional File 4).

Gene Ontology (GO) analyses were used to identify functional relationships among the various groupings of transcripts discussed above (Additional File 1). The 94 transcripts up-regulated (>1.5-fold, Benjamini-Hochberg corrected p < 0.01) in response to 1.25 μM PCI-2050 but down-regulated (>1.5-fold, Benjamini-Hochberg corrected p < 0.01) in response to actinomycin D were of special interest. These transcripts could provide insights into cellular responses to PCI-2050 that are not the result of general transcriptional inhibition. Virtually all the enriched GO categories involved transcription, chromosome organization, or RNA processing and transport (Additional File 1). Intriguingly, there were at least eight genes with transcriptional repressor activity, at least eleven known or suspected chromatin remodeling genes, and eight known or suspected RNA processing and transport genes (Table 1).

**Table 1:** Selected transcripts up-regulated by 1.25uM PCI-2050 but down-regulated by 5 ug/ml actinomycin D.

Type	Probe Set Id^a^	Gene Title^b^	Symbol	GeneID^c^	FC PCI-2050^d^	P^e^	FC ActD^f^	P^e^
Transcriptional Repressor Activity	221230_s_at	AT rich interactive domain 4B	*ARID4B*	51742	1.76	0.0067	-6.83	0.0024
	231292_at	E1A-like inhibitor of differentiation 3	*EID3*	493861	5.51	0.0006	-3.84	0.0096
	203643_at	Ets2 repressor factor	*ERF*	2077	1.74	0.0014	-3.27	0.0023
	202484_s_at	methyl-CpG binding domain protein 2	*MBD2*	8932	1.50	0.0086	-1.62	0.0038
	220746_s_at	receptor associated protein 80	*RAP80*	51720	1.60	0.0025	-1.95	0.0026
	218166_s_at	remodeling and spacing factor 1	*RSF1*	51773	2.17	0.0028	-2.78	0.0040
	219682_s_at	T-box 3 (ulnar mammary syndrome)	*TBX3*	6926	1.76	0.0043	-4.19	0.0014
	214714_at	zinc finger protein 394	*ZNF394*	84124	1.70	0.0033	-3.18	0.0012

Chromatin Remodelling	208445_s_at	bromodomain adjacent to zinc finger domain, 1B	*BAZ1B*	9031	1.62	0.0017	-2.22	0.0015
	208686_s_at	bromodomain containing 2	*BRD2*	6046	2.35	0.0008	-4.10	0.0008
	225026_at	chromodomain helicase DNA binding protein 6	*CHD6*	84181	1.72	0.0039	-2.28	0.0064
	225357_s_at	INO80 complex homolog 1 (S. cerevisiae)	*INOC1*	54617	1.95	0.0026	-2.48	0.0045
	243003_at	Myeloid/lymphoid leukemia; translocated to, 10	*MLLT10*	8028	1.61	0.0032	-2.01	0.0100
	1563321_s_at	Myeloid/lymphoid leukemia; translocated to, 10	*MLLT10*	8028	1.81	0.0071	-2.39	0.0046
	201703_s_at	protein phosphatase 1, regulatory subunit 10	*PPP1R10*	5514	1.81	0.0027	-3.88	0.0013
	209486_at	disrupter of silencing 10	*SAS10*	57050	2.02	0.0015	-5.61	0.0012
	225655_at	ubiquitin-like, with PHD & RING finger domains, 1	*UHRF1*	29128	1.56	0.0040	-1.63	0.0095
	1554441_a_at	wings apart-like homolog (Drosophila)	*WAPAL*	23063	1.67	0.0037	-3.00	0.0029
	212267_at	wings apart-like homolog (Drosophila)	*WAPAL*	23063	2.47	0.0017	-4.81	0.0033

RNA Binding and Processing	225694_at	Cdc2-related kinase, arginine/serine-rich	*CRKRS*	51755	1.51	0.0023	-2.96	0.0070
	218092_s_at	HIV-1 Rev binding protein	*HRB*	3267	1.54	0.0074	-1.64	0.0070
	223295_s_at	LUC7-like (S. cerevisiae)	*LUC7L*	55692	1.82	0.0020	-1.87	0.0032
	203378_at	PCF11, cleavage and polyadenylation factor subunit	*PCF11*	51585	2.36	0.0015	-3.89	0.0020
	238122_at	RNA binding motif protein 12B	*RBM12B*	389677	2.75	0.0010	-2.61	0.0013
	51228_at	RNA binding motif protein 12B	*RBM12B*	389677	1.81	0.0022	-2.63	0.0078
	201586_s_at	splicing factor proline/glutamine-rich	*SFPQ*	6421	1.51	0.0044	-1.77	0.0020
	217833_at	synaptotagmin binding, cytoplasmic RNA interacting	*SYNCRIP*	10492	1.52	0.0083	-1.54	0.0096
	203519_s_at	UPF2 regulator of nonsense transcripts	*UPF2*	26019	1.60	0.0059	-1.90	0.0080

^a^Affymetrix designation

^b^Abbreviated gene description

^c^NCBI GeneID designation

^d^Fold change in response to 1.25uM PCI-2050

^e^Benjamini-Hochberg corrected Student's t-test

^f^Fold change in response to 5ug/mL actinomycin D

Gene Ontology (GO) analyses of the 156 transcripts that were up-regulated in response to both dosages of PCI-2050 (Additional File 2) also were performed to investigate cellular responses to this sapphyrin. Intriguingly, RNA processing and transport genes were enriched in this group of transcripts (Additional File 1). These genes include *HNRPD *(aka *AUF1*), *CPSF6*, *SFRS4 *(aka *SRP75*), *ZNF638 *(aka *NP220*), *PCF11*, *RBPMS*, *MSI2*, IGF2BP1 (aka *CRDBP*), and *RBM16*. The induction of the putative RNA processing protein *CROP *(cisplatin resistance-associated overexpressed protein) is especially interesting since it is also over-expressed in response to the anti-cancer agent cisplatin [18]. Overall, the consistent induction of RNA processing and transport genes by both dosages of PCI-2050 highlights their importance as either potential survival responses or active cellular processes mediating the widespread reduction in transcript levels. In either case, the gene expression profiles indicate that PCI-2050 and actinomycin likely have different mechanisms of action. However, further functional analyses will need to be conducted in order to identify the molecular target(s) of sapphyrins leading to this activity.

### Single dose toxicity studies

Lethality following a single-dose intravenous injection of sapphyrin congeners was observed at doses ranging from approximately 20 μmol/kg for PCI-2012 to 75 μmol/kg for sapphyrin PCI-2042 (Table 2). Interestingly, compounds bearing an increasing number of hydroxyl group substituents were progressively better tolerated, i.e., PCI-2050 ~ PCI-2012 < PCI-2022 < PCI-2042.

**Table 2:** Single-dose toxicity in CD1 mice.

Compound	Dose (μmol/kg)	Observed Lethality
PCI-2012	20	1/3
	30	0/3
	40	3/3
		
PCI-2022	30	0/3
	40	0/3
	60	3/3
		
PCI-2050	20	0/3
	25	0/3
	30	3/3
		
PCI-2042	50	1/3
	60	0/3
	75	2/3

### Efficacy studies in a tumor xenograft model

**Figure 4:**
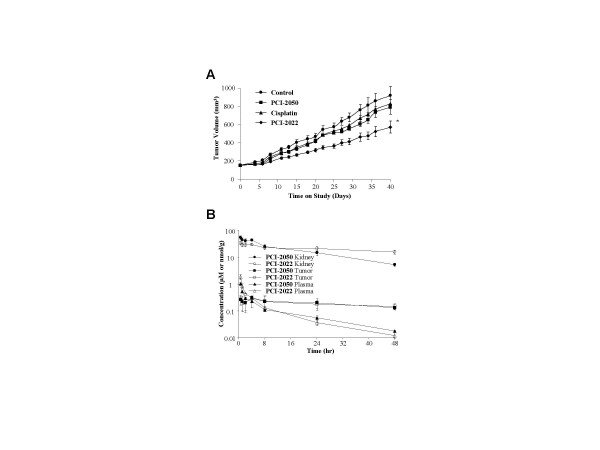
Activity of sapphyrins in A549 tumor xenograft model. *A*, Median tumor volume (± SEM) over time in mice treated with control (5% mannitol) vehicle, cisplatin (4 mg/kg q.d. × 4 i.p.), PCI-2050 (10 μmol/kg qd × 2 i.v.), or PCI-2022 (20 μmol/kg qd × 2 i.v.). * p < 0.001. *B*, Log concentration of PCI-2022 and PCI-2050 in plasma (μM) or in kidney and tumor (nmol/g) over time is shown.

Sapphyrins PCI-2050 (10 μmol/kg/day for 2 days) and PCI-2022 (20 μmol/kg/day for 2 days) were administered intravenously to A549 tumor-bearing mice (Fig. 4A). Sapphyrin doses were chosen in this case to be less than or equal to half of the maximum tolerated dose as determined by single dose toxicity studies (Table 2). Tumor growth inhibition was compared with that of cisplatin (4 mg/kg q.d. × 4 i.p.). Sapphyrin PCI-2050 and cisplatin cohorts both appeared to inhibit A549 tumor growth relative to control, although neither cohort reached statistical significance (p > 0.05). Treatment with PCI-2022 at the higher dose displayed a significant effect (p < 0.001). Weight loss of mice following treatment was less than 5% in all groups.

### Pharmacokinetic studies

The biodistribution of sapphyrins PCI-2022 and PCI-2050 in plasma, kidneys, and A549 tumor xenografts was measured over the course of 48 hours following intravenous co-administration of the compounds at a dose of 5 μmol/kg each (Fig. 4B). Plasma levels of PCI-2022 were initially higher than those of PCI-2050, but lower after 24 and 48 hour time intervals. Conversely, the levels of PCI-2050 in kidney were initially higher than those of PCI-2022, but lower after 24 and 48 hour time intervals. Tumor levels of both compounds were found to be similar after all time intervals examined.

## Discussion

Based on previous observations of the selective accumulation of sapphyrins in tumors [8], and their potent antiproliferative and cytotoxic activities [10], a series of structurally related sapphyrins was prepared and analyzed for biological activity in cell culture and xenograft model systems [11]. Initial work comparing compounds differing in the identity of solubilizing substituents (ether vs. hydroxyl) indicated that the former (PCI-2050) was superior to the hydroxylated congeners (PCI-2012, PCI-2022, PCI-2042) in terms of antiproliferative effects (Fig. 1). Further studies in vitro similarly revealed an inverse correlation between cytotoxic activity (Fig. 2B) and the number of hydroxyl group substituents present on the periphery of the sapphyrin macrocycle. These properties, perhaps unsurprisingly, were further correlated with the rate of cellular uptake of these compounds (Fig. 2C). This finding led us to conclude that the presence and identity of the solubilizing groups were relatively unimportant in terms of sapphyrin activity once the compounds penetrated the subcellular compartment. Further mechanistic studies were therefore conducted using PCI-2050, the derivative which showed the highest in vitro activity and rate of cellular uptake.

There is increasing appreciation that the induction of oxidative stress contributes to the specific and non-specific activities of a variety of anticancer agents such as bleomycin and adriamycin [19,20]. Here, we found dose-dependent formation of reactive oxygen species (ROS) occurred as an early event in A549 cells treated with PCI-2050 (Fig. 2D). The formation of ROS is consistent with the relative ease of reduction of this expanded porphyrin [5], which could lead to redox cycling under biological conditions. This effect appeared at a similar time and concentration required to inhibit 5-bromo-2'-deoxyuridine incorporation and cellular proliferation (Fig. 2A), prior to cell death. Treatment with actinomycin D (5 μg/mL = 4 μM) also led to an increase of ROS (2.3-fold), although not to the same degree as the corresponding molar equivalent of PCI-2050. In terms of potency, sapphyrin activity appeared similar to and in some cases (e.g., PCI-2050) superior to that of cisplatin, but lower than that of actinomycin D (Cf., Figs. 1 and 2A).

In an effort to gain a more detailed understanding of the mechanistic basis for its anticancer activity, we studied the effect of PCI-2050 on gene expression profiles in plateau phase A549 cells. We selected plateau phase cultures in order to focus on expression changes occurring in response to treatment with drug that are not effected by cell cycle occupancy. In preliminary studies, we observed widespread decreases in transcript levels within four hours of exposure to two different dosages of PCI-2050. In a global sense, this mimicked cellular responses to actinomycin D, a well-characterized transcriptional inhibitor. However, several striking differences in gene expression profiles (Fig. 3, Table 1) between these two anti-cancer agents highlight the fact that they likely promote wide-spread transcript decreases by unrelated mechanisms. For example, the induction of transcription factors with known repressor activities, chromatin remodeling genes, and RNA processing and transport transcripts by the lower dose of PCI-2050 contrasts their down-regulation in response to actinomycin D (Table 1). RNA processing and transport transcripts were up-regulated in response to both dosages of PCI-2050 (Additional Files 1 and 2). We propose that the induction of these transcripts serve as survival responses aimed at countering the effects of drug-induced transcript decreases. This information provides insights into biochemical pathways which regulate cellular transcriptional activity on a global-scale.

The widespread decreases in transcript levels due to PCI-2050 observed by microarray are consistent with the results of COMPARE analysis of antiproliferative activity in the NCI 60 tumor line panel, which includes PC3 cells, indicating a similarity to anticancer agents known to inhibit RNA transcription or protein synthesis. The COMPARE algorithm does not appear to distinguish between compounds targeting these two processes [14]. Overall, the mechanisms of other agents that produce wide-spread decreases in transcript levels such as actinomycin D, 5,6-dichloro-1-h-ribofuranosylbenzimidazole (DRB), flavopiridol, 4-amino-6-hydrazino-7-beta-D-ribofuranosyl-7H-pyrrolo(2,3-d)-pyrimidine-5-carboxamide (ARC), and α-amanatin are quite varied. For example, actinomycin D is thought to inhibit transcription initiation by intercalating into DNA [21]. In contrast, DRB, flavopiridol, and ARC inhibit transcription elongation by affecting the activity of factor P-TEFb, a complex of cyclins with CDK9 [22,23]. Meanwhile, α-amanitin directly inhibits both transcriptional initiation and elongation by binding directly to RNA polymerase II [24]. In addition, the topoisomerase inhibitor camptothecin has also been reported to affect wide-spread reductions in transcript levels in cultured cancer cells [25]. Thus, multiple nucleic acid metabolic pathways can be targeted to yield wide-spread decreases in transcript levels. Although it could prove challenging to identify the specific molecular mechanism mediating sapphyrin activity, there are several powerful experimental approaches to address this issue. For example, nuclear run-on assays and immunoblot analyses of phospho-RNA polymerase II levels have proven valuable for the dissecting the molecular basis for ARC activity [22,23]. In addition, the affinity of sapphyrin for DNA, RNA, and various proteins, especially those involved in nucleic acid structure and function, can be tested by affinity chromatography or surface plasmon resonance (SPR) based techniques. Furthermore, it is possible that genetic screens for sapphyrin-resistance in model organisms (e.g. yeast) could shed light on its molecular targets.

Although the precise molecular target for their effects remains undefined, the identification of sapphyrins as general transcriptional inhibitor, based on COMPARE analysis of the NCI 60 tumor line panel and A549 gene expression profiling experiments, provides a plausible mechanism for their anticancer activity. Inhibition of gene expression can effectively squelch transcriptional responses to stresses and compromise cellular survival responses. For example, batteries of survival genes downstream of the transcription factors MTF-1, HIF-1, and NRF-2 previously shown to be induced in A549 cells under oxidative stress were not differentially expressed in response to PCI-2050 or actinomycin D, despite ROS accumulation [26,27]. Additional *in vitro *studies are required to determine whether transcriptional inhibition is causally related to the formation of ROS, and, if so, to delineate the pathways involved. It is also important to conduct similar studies *in vivo *to confirm that conclusions derived from cultured cancer cells are valid in whole organisms.

PCI-2050 was previously shown to display significant antitumor activity in Ramos B-cell lymphoma and RKO colon cancer xenograft models [11]. In this study, we explored the effect of different solubilizing substituents on in vitro activity and compared two compounds in a somewhat more resistant A549 lung cancer xenograft model. Although PCI-2050 displayed the highest biological activity in vitro, compounds substituted with multiple hydroxyl groups, such as sapphyrin PCI-2022, appeared to be better tolerated (Table 2), and may therefore offer improved therapeutic potential. For example, comparison of PCI-2050 and PCI-2022, administered at similarly well-tolerated doses (10 or 20 μmol/kg, respectively, for two days), indicated that a significant benefit (p < 0.001) could be obtained using the higher dose of the better-tolerated compound (Fig. 4A).

To assess whether pharmacokinetic properties could account for these differences, the biodistributions of PCI-2050 and PCI-2022 in plasma, kidney, and tumor were compared by administering equimolar amounts of these compounds in a single-dose injection. Although it is possible that drug-drug interactions might interfere with the absolute pharmacokinetic parameters obtained in this study, this approach provides an internally controlled assessment of the relative distribution of these two agents. In fact, in replicate measurements, the ratio of the biological concentrations of the two compounds is more precise than their individual concentrations (compare Additional File 1 items S7 to S10). These data indicate the levels of PCI-2050 and PCI-2022 in A549 xenograft tumors were similar after all times intervals examined (30 minutes to 48 hours), consistent with similar levels of tumor growth inhibition for the two agents. On the other hand, after early time intervals, the ratio of PCI-2050 to PCI-2022 was higher in kidney, the presumed organ of toxicity, based on histological studies (data not shown). These findings lead us to suggest distribution as a possible explanation for the improved tolerance of PCI-2022 relative to PCI-2050.

## Conclusion

Sapphyrins inhibit the proliferation of adherent cancer cell lines *in vitro *at relatively low doses, leading to cell death within a few days. Treatment A549 cells with sapphyrin PCI-2050 elicits the formation of ROS and results in wide-spread decreases in transcript levels within four hours. This inhibition is accompanied by a dosage-dependent induction of RNA processing and transport, transcriptional repressor, and chromatin remodeling genes. These gene expression profiles dramatically differ from those associated with other anticancer agents, such as motexafin gadolinium, that induce oxidative stress in cancer cells. In our *in vivo *studies, mice better tolerated water-soluble sapphyrin analogues bearing multiple hydroxyl groups relative to those with amphipathic functionalities. Sapphyrin PCI-2022 inhibited A549 tumor growth significantly in a xenograft model using a reasonably well tolerated dosing regimen. Thus, hydrophilic sapphyrin analogues display promise as investigational new drug candidates for the treatment of solid tumors.

## Methods

### Cells and cell culture reagents

A549 lung and PC3 prostate cancer cell lines were purchased from the American Type Culture Collection. Unless otherwise indicated, all cell culture reagents were purchased from Invitrogen (Carlsbad, CA). Cells were cultured in a 5% CO_2 _incubator at 37°C in RPMI 1640 medium supplemented with 20 mM HEPES, 2 mM L-glutamine, 10% fetal bovine serum (Biomeda, Foster City, CA) and antibiotics (200 U/mL penicillin and 200 μg/mL streptomycin) as previously described [26]. Cisplatin (Sigma Chemical) was formulated in 0.9% saline and stored at -20°C prior to use.

### Cell viability

Cell viability was determined by using propidium iodide (PI) flow cytometric analysis. A549 cells (2.5 × 10^5 ^cells per plate) were seeded in 10 cm plates and allowed to adhere overnight prior to treatment with sapphyrins or actinomycin D. After 24 hours, the drug-containing medium was removed, fresh medium was added, and the cultures were allowed to grow an additional 48 hours. To harvest, cells were washed with phosphate buffered saline (PBS), and treated with 0.025% trypsin/EDTA for 10 minutes. Complete medium was then added and the cells isolated by centrifugation. Cells present in the growth medium and wash solution were isolated by centrifugation and included in the analysis. Cells were re-suspended in 1 mL 10% bovine serum albumin (BSA) in PBS, an aliquot of 3 × 10^5 ^cells transferred to a 4 mL tube, and the cells isolated by centrifugation. Cell pellets were re-suspended in 10% BSA in PBS supplemented with 2 μg/mL propidium iodide (Sigma), incubated for 5 minutes at ambient temperature, and subjected to flow cytometric analysis. Flow cytometry was performed on a FACSCalibur instrument and data were analyzed using the CellQuest Pro software package (BD Biosciences).

### Cell proliferation

The proliferation of exponential phase cultures of A549 and PC3 cells was assessed by tetrazolium salt reduction [28]. In brief, A549 (2000 cells/well) or PC3 (4000 cells/well) cells were seeded on 96-well microtiter plates and allowed to adhere overnight. Stock solutions of sapphyrins or other inhibitors in medium were added by serial dilution and plates were incubated at 37°C under a 5% CO_2_/95% air atmosphere. After 24 hours, medium was replaced with fresh medium. After 2 additional days, medium was exchanged for fresh medium (150 μL/well) supplemented with 3-(4,5-dimethylthiazol-2-yl)-2,5-diphenyltetrazolium bromide (MTT, Sigma Chemical, 0.5 mg/mL). Plates were incubated at 37°C for two hours and viable cells measured as described [26].

### Measurement of reactive oxygen species

Reactive oxygen species (ROS) were measured in live cells as intracellular peroxides by monitoring the oxidation of 2',7'-dichlorofluorescin-diacetate (DCFA) to 2',7'-dichlorofluorescein (DCF) (Invitrogen). Cultures were harvested as described above. Cells (1 × 10^6 ^per mL) were incubated in a solution of 1 μg per mL DCFA in 0.5% BSA in HBSS for 15 minutes at 37°C. Two mL additional 0.5% BSA in HBSS was added, cells were isolated by centrifugation, and the pellet re-suspended in a solution of 50 μg/mL 7-aminoactinomycin D (7-AAD) in 0.5% BSA in HBSS. Cell suspensions were incubated at ambient temperature for 2 to 3 minutes, and stored on ice until analysis. The fluorescent intensity in live (i.e., 7-AAD impermeable) cells was analyzed by flow cytometry as described [27].

### Measurement of DNA synthesis

Exponential phase A549 cultures were treated with control 5% mannitol vehicle or sapphyrin PCI-2050 for 4, 8, or 31 hours. Thirty minutes prior to harvest, cultures were treated with 5-bromo-2'-deoxyuridine (BrdU) at a final concentration of 10 μM. Cells were isolated as described above, washed once with 0.5% BSA in PBS, and the resulting cell pellets fixed using 0.5 mL Cytofix/Cytoperm reagent (BD Biosciences). After incubation at ambient temperature for 30 minutes, cells were isolated by centrifugation, washed with 3% FBS in PBS, re-suspended in 10% DMSO in medium, and stored at -20°C until analysis. Cells were stained using a fluorescein-conjugated anti-BrdU antibody (clone PRB1, E-Bioscience, San Diego, CA) and 7-AAD. Cell cycle occupancy was analyzed by flow cytometry using fluorescein signal as a measure of DNA synthesis and 7-AAD signal as a measure of DNA content as described [29]. For comparison, cultures were treated with actinomycin D (200 ng/mL).

### National Cancer Institute cell line screen

Testing of PCI-2050 in the NCI Developmental Therapeutics Program panel of 60 human tumor cell lines yielded an overall log value of drug concentration leading to 50% growth inhibition (Log GI_50_) of -7.04 with a range of 2.34 (Additional File 1). Log GI_50 _was determined after 48 hours treatment using the sulforhodamine B assay. Individual cell line values of Log GI_50 _were uploaded manually into the COMPARE program website [30] to generate a seed data set. This was analyzed using the COMPARE algorithm [13] for correlation with mean graph patterns of compounds (over 40,000) present in the synthetic compound database. Analysis was performed using default program parameters.

### Gene expression profiling

A549 human lung cancer cells (1 × 10^5 ^cells per T-25 flask in 7 mL complete RPMI 1640 medium) were seeded eight days prior to treatment of non-cycling plateau phase cultures with drug. At four hours prior to RNA isolation, sapphyrin PCI-2050 (1.25 or 2.5 μM final concentration), actinomycin D (5 μg/mL final concentration), or control (5% mannitol) solution was added to the cultures. Each experiment was performed in triplicate. After incubation, all cultures were washed twice with HBSS supplemented with 0.5% BSA and total RNA was isolated and subjected to analysis on Human Genome U133 Plus 2.0 Arrays (Affymetrix, Santa Clara, CA), as described [31]. ArrayAssist software (Stratagene/Affymetrix) and the RMA (Robust Microarray Analysis) algorithm were used to generate scaled gene expression values. Genes at least 1.5-fold differentially expressed in drug-treated relative to control samples with a Benjamini-Hochberg corrected Student's t-test p = 0.01 are reported. GeneOntology analyses were conducted using WebGestalt software [32,33]. All scaled fluorescent intensity values and .cel files are available at the National Center for Biotechnology Information (NCBI) Gene Expression Omnibus (GEO) repository [34] under Series Accession Number GSE6400. In addition, all scaled fluorescent intensity values are available in Additional File 2.

### Mouse xenograft model

Animal care was in accordance with NIH and institutional guidelines. For the A549 xenograft model [35], 1.25 × 10^6 ^A549 cells were injected subcutaneously/intramuscularly into the right hind flank of 6 week old CD-1 nude mice that had been irradiated with 4 Gy of total body irradiation from a ^137^Cs radiation source one day prior to tumor implantation. When the average size of tumors reached approximately 100 mm^3^, mice were randomized by tumor size to treatment groups containing 6 mice per group. Mice were treated intravenously with 2 doses of sapphyrin, 10 or 20 μmol/kg, on consecutive days. Vehicle control treated animals received 5% mannitol on the same schedule. Tumor and body weight measurements were performed three times per week. Tumor volume was calculated using the equation V (mm^3^) = a × b^2^/2, where a is the largest diameter and b is the smallest diameter. Data shown represent the mean ± standard error of mean (± SEM). Body weight loss was less than 5% for all compounds tested. One-way ANOVA on log transformed data was performed using GraphPad Prism (GraphPad Software Inc., San Diego, CA). P-values were corrected for multiple comparisons to control using Dunnett's test.

### Pharmacokinetic measurements

Female A549 tumor-bearing CD1 nude mice were prepared as described above. When the average size of tumors reached approximately 500 mm^3^, mice were randomly assigned to study groups according to their tumor volume on day 1 and then administered a single intravenous injection of control vehicle (pre-dose) or a mixture of two sapphyrins (5 μmol/kg PCI-2022 and 5 μmol/kg PCI-2050 for a total sapphyrin dose of 10 μmol/kg). Blood samples for pharmacokinetic analysis were obtained by cardiac puncture into lithium heparin at the following time points: 0 (pre-dose), 0.5, 1, 2, 4, 8, 24, and 48 hours post injection. The plasma was separated by centrifugation and stored frozen until analysis.

Tumor and kidney were collected from each animal immediately following the euthanasia procedure to evaluate the biodistribution of the compound. Plasma and selected tissue samples were analyzed for PCI-2022 and PCI-2050 using an HPLC method with fluorescence detection as described in the Additional File 1.

## Competing interests

The following relationships could be construed as resulting in an actual, potential, or apparent conflict of interest with regard to the manuscript submitted for review: Several authors are employees of Pharmacylics, Inc., the company that is developing sapphyrins. Moreover, the Hacia and Sessler laboratories have received research support from Pharmacyclics.

## Authors' contributions

ZW, CC, and D-G C contributed to compound design, synthesis, and/or characterization. PSL performed flow cytometry analyses and obtained samples for microarray analysis. PT and QF designed tumor growth studies and/or toxicity studies. XM performed tumor growth studies and toxicity studies. DT and DM designed bioanalytical methods and/or pharmacokinetic experiments. LN obtained NCI60 cell line data and contributed to writing the manuscript. RAM contributed to writing the manuscript. DMagda performed cellular proliferation assays, contributed to designing compounds, experiments, and writing the manuscript. JLS contributed to compound design and writing the manuscript. BLP, SMY, and MWK acquired and analyzed gene expression data. JGH contributed to designing experiments, writing the manuscript, and conducting the gene expression data analyses.

## Supplementary Material

Additional file 1 Methods and statistical analyses used in these studies. Here we provide Gene Ontology (GO) analyses, the protocol for tissue extraction methodology and supplemental biodistribution analyses, details concerning the synthesis of hydrophilic sapphyrins, and NCI Developmental Therapeutics Program tumor cell line panel data Click here for file

Additional file 2 Normalized hybridization signal intensity values for all microarray experiments. This file provides all the processed hybridization signals generated in gene expression profiling experiments along with statistical analyses of differentially expressed genes. Click here for file

Additional file 3 Selected gene expression data Part I. Data from all 94 probe tilings showing up-regulation in response to 1.25uM sapphyrin PCI-2050 and down-regulation in response to 5ug/mL actinomycin D Click here for file

Additional file 4 Selected gene expression data Part II. Probe tilings up-regulated by both doses of PCI-2050 but down-regulated by actinomycin D. Click here for file

## References

[B1] Bauer VJ, Clive DLJ, Dolphin D, Paine JB, Harris FL, King MM, Loder J, Wang SWC, Woodward RB (1983). Sapphyrins:  Novel Aromatic Pentapyrrolic Macrocycles. j am chem soc.

[B2] Broadhurst MJ, Grigg R, Johnson AW (1972). Synthesis of 22-Pi-Electron Macrocycles, Sapphyrins and Related Compounds.. J Chem Soc, Perkin Trans.

[B3] Sessler J, Cyr MJ, Lynch V, McGhee E, Ibers JA (1990). Synthetic and Structural Studies of Sapphyrin, a 22-Pi-Electron Pentapyrrolic "Expanded Porphyrin". j am chem soc.

[B4] Sessler JL, Davis JM (2001). Sapphyrins: versatile anion binding agents. Acc Chem Res.

[B5] Springs SL, Gosztola D, Wasielewski M, Kral V, Andreivsky A, Sessler JL (1999). Picosecond Dynamics of Energy Transfer in Porphyrin-Sapphyrin Noncovalent Assemblies. j am chem soc.

[B6] Judy MM, Matthews JL, Newman JT, Skiles HL, Boriack RL, Sessler JL, Cyr M, Maiya BG, Nichol ST (1991). In vitro photodynamic inactivation of herpes simplex virus with sapphyrins: 22 pi-electron porphyrin-like macrocycles. Photochem Photobiol.

[B7] Maiya BG, Cyr M, Harriman A, Sessler JL (1990). Photodynamic Activity of Diprotonated Sapphyrin:  A 22-Pi-Electron Pentapyrrolic Porphyrin-like Macrocycle. j phys chem.

[B8] Kral V, Davis J, Andrievsky A, Kralova J, Synytsya A, Pouckova P, Sessler JL (2002). Synthesis and biolocalization of water-soluble sapphyrins. J Med Chem.

[B9] Kral V, Brucker EA, Hemmi G, Sessler JL, Kralova J, Bose H (1995). A non-ionic water-soluble pentaphyrin derivative. Synthesis and cytotoxicity. Bioorg Med Chem.

[B10] Naumovski L, Ramos J, Sirisawad M, Chen J, Thiemann P, Lecane P, Magda D, Wang Z, Cortez C, Boswell G, Gyu CD, Sessler J, Miller R (2005). Sapphyrins induce apoptosis in hematopoietic tumor-derived cell lines and show in vivo antitumor activity. Mol Cancer Ther.

[B11] Naumovski L, Sirisawad M, Lecane P, Chen J, Ramos J, Wang Z, Cortez C, Magda D, Thiemann P, Boswell G, Miles D, Cho DG, Sessler JL, Miller R (2006). Tumor localization and antitumor efficacy of novel sapphyrin compounds. Mol Cancer Ther.

[B12] Rosenkranz AR, Schmaldienst S, Stuhlmeier KM, Chen W, Knapp W, Zlabinger GJ (1992). A microplate assay for the detection of oxidative products using 2',7'-dichlorofluorescin-diacetate. J Immunol Methods.

[B13] Zaharevitz DW, Holbeck SL, Bowerman C, Svetlik PA (2002). COMPARE: a web accessible tool for investigating mechanisms of cell growth inhibition. J Mol Graph Model.

[B14] Chan J, Khan SN, Harvey I, Merrick W, Pelletier J (2004). Eukaryotic protein synthesis inhibitors identified by comparison of cytotoxicity profiles. RNA.

[B15] Zalacain M, Zaera E, Vazquez D, Jimenez A (1982). The mode of action of the antitumor drug bouvardin, an inhibitor of protein synthesis in eukaryotic cells. FEBS Lett.

[B16] Simonova VS, Samusenko AV, Filippova NA, Tevyashova AN, Lyniv LS, Kulik GI, Chekhun VF, Shtil AA (2005). Olivomycin induces tumor cell apoptosis and suppresses p53-induced transcription. Bull Exp Biol Med.

[B17] Yang E, van Nimwegen E, Zavolan M, Rajewsky N, Schroeder M, Magnasco M, Darnell JE (2003). Decay rates of human mRNAs: correlation with functional characteristics and sequence attributes. Genome Res.

[B18] Umehara H, Nishii Y, Morishima M, Kakehi Y, Kioka N, Amachi T, Koizumi J, Hagiwara M, Ueda K (2003). Effect of cisplatin treatment on speckled distribution of a serine/arginine-rich nuclear protein CROP/Luc7A. Biochem Biophys Res Commun.

[B19] Brend N (1985). Inhibition of bleomycin lung toxicity by n-acetyl cysteine in the rat. Pathology.

[B20] Sinha BK, Mimnaugh EG, Rajagopalan S, Myers CE (1989). Adriamycin activation and oxygen free radical formation in human breast tumor cells: protective role of glutathione peroxidase in adriamycin resistance. Cancer Res.

[B21] Chen H, Liu X, Patel DJ (1996). DNA bending and unwinding associated with actinomycin D antibiotics bound to partially overlapping sites on DNA. J Mol Biol.

[B22] Radhakrishnan SK, Gartel AL (2006). A novel transcriptional inhibitor induces apoptosis in tumor cells and exhibits antiangiogenic activity. Cancer Res.

[B23] Lam LT, Pickeral OK, Peng AC, Rosenwald A, Hurt EM, Giltnane JM, Averett LM, Zhao H, Davis RE, Sathyamoorthy M, Wahl LM, Harris ED, Mikovits JA, Monks AP, Hollingshead MG, Sausville EA, Staudt LM (2001). Genomic-scale measurement of mRNA turnover and the mechanisms of action of the anti-cancer drug flavopiridol. Genome Biol.

[B24] Khobta A, Ferri F, Lotito L, Montecucco A, Rossi R, Capranico G (2006). Early effects of topoisomerase I inhibition on RNA polymerase II along transcribed genes in human cells. J Mol Biol.

[B25] Carson JP, Zhang N, Frampton GM, Gerry NP, Lenburg ME, Christman MF (2004). Pharmacogenomic identification of targets for adjuvant therapy with the topoisomerase poison camptothecin. Cancer Res.

[B26] Magda D, Lecane P, Miller RA, Lepp C, Miles D, Mesfin M, Biaglow JE, Ho VV, Chawannakul D, Nagpal S, Karaman MW, Hacia JG (2005). Motexafin gadolinium disrupts zinc metabolism in human cancer cell lines. Cancer Res.

[B27] Lecane PS, Karaman MW, Sirisawad M, Naumovski L, Miller RA, Hacia JG, Magda D (2005). Motexafin gadolinium and zinc induce oxidative stress responses and apoptosis in B-cell lymphoma lines. Cancer Res.

[B28] Mosmann T (1983). Rapid colorimetric assay for cellular growth and survival: application to proliferation and cytotoxicity assays. J Immunol Methods.

[B29] Gratzner HG, Leif RC (1981). An immunofluorescence method for monitoring DNA synthesis by flow cytometry. Cytometry.

[B30] COMPARE program website [http://dtp.nci.nih.gov/docs/compare/compare.html ].

[B31] Karaman MW, Houck ML, Chemnick LG, Nagpal S, Chawannakul D, Sudano D, Pike BL, Ho VV, Ryder OA, Hacia JG (2003). Comparative analysis of gene-expression patterns in human and African great ape cultured fibroblasts. Genome Res.

[B32] WebGestalt software [http://bioinfo.vanderbilt.edu/webgestalt/ ].

[B33] Zhang B, Kirov S, Snoddy J (2005). WebGestalt: an integrated system for exploring gene sets in various biological contexts. Nucleic Acids Res.

[B34] NCBI Gene Expression Omnibus repository [http://www.ncbi.nlm.nih.gov/geo/ ].

[B35] Nishizaki M, Meyn RE, Levy LB, Atkinson EN, White RA, Roth JA, Ji L (2001). Synergistic inhibition of human lung cancer cell growth by adenovirus-mediated wild-type p53 gene transfer in combination with docetaxel and radiation therapeutics in vitro and in vivo. Clin Cancer Res.

