# Supplementary material for: Synthesis and biologic properties of hydrophilic sapphyrins, a new class of tumor-selective inhibitors of gene expression
Source: Mol Cancer. 2007 Jan 19;6:9. doi: 10.1186/1476-4598-6-9 (PMC1784109; doi:10.1186/1476-4598-6-9)
Supplement: Additional file 1 — Methods and statistical analyses used in these studies. Here we provide Gene Ontology (GO) analyses, the protocol for tissue extraction methodology and supplemental biodistribution analyses, details concerning the synthesis of hydrophilic sapphyrins, and NCI Developmental Therapeutics Program tumor cell line panel data [file 1476-4598-6-9-S1.doc]

**Synthesis and Biologic Properties of Hydrophilic Sapphyrins, a New Class of Tumor-Selective Inhibitors of Gene Expression**

Zhong Wang,^1^ Philip S. Lecane,^1^ Patricia Thiemann,^1^ Qing Fan,^1^ Cecilia Cortez,^1^ Xuan Ma,^1^ Danielle Tonev,^1^ Dale Miles,^1^ Louie Naumovski,^1^ Richard A. Miller,^1^ Darren Magda,^1§^ Dong-Gyu Cho,^2^ Jonathan L. Sessler,^2^ Brian L. Pike,^3^ Samantha M. Yeligar,^3^ Mazen W. Karaman,^3^ and Joseph G. Hacia^3^

^1^Pharmacyclics, Inc., Sunnyvale, California; ^2^Department of Chemistry and Biochemistry, University of Texas at Austin, Austin, Texas; and ^3^Department of Biochemistry and Molecular Biology, University of Southern California, Los Angeles, California; ^§^Corresponding author

**Contents of Additional File 1**

**I Gene Ontology analyses (Pages S1-S7)**

**II Protocol for tissue extraction methodology and supplemental biodistribution analyses (Pages S8-S15)**

**III Synthesis of hydrophilic sapphyrins (Pages S16 – S17)**

**IV NCI Developmental Therapeutics Program tumor cell line panel data (Pages S18-S19)**

Supplemental Figures 1-3. Gene Ontology Analysis of Gene Expression Profiles Part I. A total of 94 transcripts were found in cultured A549 cells to be both (i) 1.5-fold up-regulated (Benjamini-Hochberg corrected Student’s t-test p≤0.01) in response to treatment for four hours with 1.25 μM PCI-2050 and (ii) 1.5-fold down-regulated (Benjamini-Hochberg corrected Student’s t-test p≤0.01) in response to treatment for four hours with 5 μg/ml actinomycin D. These identifiers for these genes were imported into WebGestalt software (http://genereg.ornl.gov/webgestalt/) for Gene Ontology Analysis. Briefly, this highlights functional categories of genes that are enriched in this dataset relative to chance. We report all categories containing at least four genes with p<0.05, correcting for the abundance of genes within a functional category in the Human Genome U133 Plus 2.0 Arrays used for these analyses. Enriched gene categories relevant to (a) Biological Process, (b) Molecular Function, and (c) Cellular Component are labeled in red.

Supplemental Figures 4-6. Gene Ontology Analysis of Gene Expression Profiles Part II. A total of 156 transcripts were found in cultured A549 cells to be 1.5-fold up-regulated (Benjamini-Hochberg corrected Student’s t-test p≤0.01) in response to treatment for four hours with 1.25 and 2.50 μM PCI-2050. These identifiers for these genes were imported into WebGestalt software for Gene Ontology Analysis. We report all categories containing at least four genes with p<0.05, correcting for the abundance of genes within a functional category in the Human Genome U133 Plus 2.0 Arrays used for these analyses. Enriched gene categories relevant to (a) Biological Process, (b) Molecular Function, and (c) Cellular Component are labeled in red.

**Figure S1.**  **BIOLOGICAL PROCESS** Transcripts were 1.5-fold up-regulated, p<0.01 in response to 1.25 μM PCI-2050 and 1.5-fold down-regulated, p<0.01 in response to treatment with 5 μg/ml actinomycin D.

**Figure S2. MOLECULAR FUNCTION.** Transcripts were 1.5-fold up-regulated, p<0.01 in response to 1.25 μM PCI-2050 and 1.5-fold down-regulated, p<0.01 in response to treatment with 5 μg/ml actinomycin D.

**Figure S3. CELLULAR COMPONENT.** Transcripts were 1.5-fold up-regulated, p<0.01 in response to 1.25 μM PCI-2050 and 1.5-fold down-regulated, p<0.01 in response to treatment with 5 μg/ml actinomycin D.

**Figure S4.**  **BIOLOGICAL PROCESS.** Transcripts were 1.5-fold up-regulated, p<0.01 in response to treatment for four hours with 1.25 and 2.50μM PCI-2050.

**Figure S5. MOLECULAR FUNCTION.** Transcripts were 1.5-fold up-regulated, p<0.01 in response to treatment for four hours with 1.25 and 2.50μM PCI-2050.

**Figure S6. CELLULAR COMPONENT** Transcripts were 1.5-fold up-regulated, p<0.01 in response to treatment for four hours with 1.25 and 2.50μM PCI-2050.

**Protocol for tissue extraction methodology and supplemental biodistribution analyses**

Summary

Female A549 tumor-bearing CD1 nude mice were randomly assigned to study groups according to their tumor volume on day 1 and then administered a single intravenous injection of a mixture of two sapphyrins (5 µmol/kg **PCI-2022** + 5 µM/kg **PCI-2050** for a total sapphyrin dose of 10.0 µmol/kg). Blood samples for pharmacokinetic analysis were obtained by cardiac puncture into lithium heparin at the following time points: 0 (pre-dose), 0.5, 1, 2, 4, 8, 24, and 48 hours post injection. The plasma was separated by centrifugation and stored frozen until analysis.

Tumor and kidney were collected from each animal immediately following the euthanasia procedure to evaluate the biodistribution of the compound. Plasma and selected tissue samples were analyzed for **PCI-2022** and **PCI-2050** using an HPLC method with fluorescence detection (see Tables S1 and S2).

Figures S4-S6 show the concentration-time data obtained from the study. Figure S7 shows the ratio of the concentration of **PCI-2050** to **PCI-2022** over time. The raw data from the study are shown in Tables S3-S5.

Bioanalytical Procedure - Plasma

Bioanalytical analysis was done by thawing samples to room temperature and adding CHAPS detergent (0.5 M stock concentration) to final concentration of approximately 16 mM in each sample, followed by immediate mixing. The volume of CHAPS stock solution added to each sample was approximately 3% of the volume of the plasma sample. All samples were processed on ice. Standards and QCs were prepared in blank lithium heparin mouse plasma containing 16 mM CHAPS detergent. Standards were prepared from a 500 µL aliquot of the dosing solution that was identical to the solution used for dosing the animals in the study. Samples were processed and then analyzed by HPLC with fluorescence detection using the conditions shown in Table S1 below. The back-calculated concentration for all standards fell within ± 15% of nominal. QC samples all fell within ± 15% of nominal.

Bioanalytical Procedure – Tumor and Kidney

All procedures were done on ice. Each tissue sample was weighed and minced. Approximately 100 mg of the minced tissue was accurately weighed into a polypropylene tube and homogenized on ice using a Polytron PT-DA 1207/2EC generator powered by a PT1200 motor (Brinkmann Instruments, Westbury, NY, USA) for 15 to 30 seconds at 15,000 RPM.. In preparation for analysis by HPLC, 100 µL of each sample was added to a microcentrifuge tube. Four microliters of 1 M Tris Buffer (pH 8) was added to each tube, followed by vortex mixing. All samples were processed on ice. For the tumor study, standards were prepared in 20 % blank kidney homogenate and QCs were prepared in 20 % blank mouse tumor homogenate. For the kidney study, standards and QCs were prepared in 20 % blank kidney homogenate. All standards and QC samples were prepared from a 500 µL aliquot of the dosing solution that was identical to the solution used for dosing the animals in the study. Samples were processed and then analyzed by HPLC with fluorescence detection using the conditions shown in Table S2 below. A set of calibration standards prepared in kidney homogenate were placed at the beginning of each run. Sample concentrations for final reporting were calculated by regression using the set of standards.

Figure S7. Concentration-time profiles in plasma for **PCI-2022** and **PCI-2050** after co-administration (5 µmol/kg/compound) to CD-1 nude mice bearing A549 tumors.

Figure S8. Concentration-time profiles in tumor for **PCI-2022** and **PCI-2050** after co-administration (5 µmol/kg/compound) to CD-1 nude mice bearing A549 tumors.

Figure S9. Concentration-time profiles in kidney for **PCI-2022** and **PCI-2050** after co-administration (5 µmol/kg/compound) to CD-1 nude mice bearing A549 tumors.

Figure S10. Ratio of concentrations of compounds **PCI-2022** and **PCI-2050** after co-administration (5 µmol/kg/compound) to CD-1 nude mice bearing A549 tumors.

Table S1. Summary of the HPLC method used to analyze PCI-2022 and PCI-2050 in mouse plasma.

| **General Information** | **Description** |
| --- | --- |
| Analyte | PCI-2050, PCI-2022 |
|  |  |
| **Sample Processing** | **Description** |
| Volume of Sample (Containing 16 mM CHAPS detergent) | 20 µL |
| Volume of 40% Zinc Sulfate in Water | 2 µL |
| Volume of 50 µM MGd (IS) | 2 µL |
| Volume of Precipitating Solution (50/50 v/v MeOH/ACN w/ 0.16 M acetic acid ) | 20 µL |
|  |  |
| **HPLC Conditions** | **Description/Setting** |
| HPLC System | Agilent 1100 Integrated HPLC System |
| Column | Zorbax Eclipse SB-CN (3.0 x 150 mm, 3.5 µm particle size) |
| Mobile Phase | | Time (min) | Flow Rate (mL/min) | % Solvent B | % Solvent C | | --- | --- | --- | --- | | 0.00 | 0.950 | 24 | 76 | | 3.00 | 0.950 | 35 | 65 | | 12.00 | 0.950 | 60 | 40 | | 13.00 | 0.950 | 24 | 76 | | 15.00 | 0.950 | 24 | 76 | |
| Injection Volume | 15 µL |
| Elution type | Gradient |
| Flow rate | 0.950 mL/min |
| Column temperature  Autosampler temperature | 55 °C  4 °C |
| Detection | Fluorescence with excitation 436 nm (with Schott BG-39 excitation filter) and emission at 750 nm (using an Andover emission filter). |
|  |  |

Table S2. Summary of the HPLC method used to analyze PCI-2022 and PCI-2050 in mouse kidneys and tumors.

| **General Information** | **Description** |
| --- | --- |
| Analyte | PCI-2050, PCI-2022 |
|  |  |
| **Sample Processing** | **Description** |
| Volume of Sample | 100 µL |
| Volume of 1 M Tris buffer, pH 8.0 | 4 µL |
| Volume of 40% Zinc Sulfate in Water | 10 µL |
| Volume of 100 µM MGd (IS) | 10 µL |
| Volume of Precipitating Solution (50/50 v/v MeOH/ACN | 100 µL |
|  |  |
| Volume glacial acetic acid (added to supernatant after transferring) | 4 µL |
| **HPLC Conditions** | **Description/Setting** |
| HPLC System | Agilent 1100 Integrated HPLC System |
| Column | Zorbax Eclipse SB-CN (3.0 x 150 mm, 3.5 µm particle size) |
| Mobile Phase | | Time (min) | Flow Rate (mL/min) | % Solvent B | % Solvent C | | --- | --- | --- | --- | | 0.00 | 0.950 | 24 | 76 | | 3.00 | 0.950 | 35 | 65 | | 12.00 | 0.950 | 60 | 40 | | 13.00 | 0.950 | 24 | 76 | | 15.00 | 0.950 | 24 | 76 | |
| Injection Volume | 15 µL |
| Elution type | Gradient |
| Flow rate | 0.950 mL/min |
| Column temperature  Autosampler temperature | 55 °C  4 °C |
| Detection | Fluorescence with excitation 436 nm (with Schott BG-39 excitation filter) and emission at 750 nm (using an Andover emission filter). |
|  |  |

Table S3. Concentrations of PCI-2022 and PCI-2050 determined in lithium heparin mouse plasma.

| Sample ID | Time Post Injection (hr) | Final Conc. Of PCI-2022 (µM) | Mean | StDev | RSD | Final Conc. Of PCI-2050 (µM) | Mean | StDev | RSD |
| --- | --- | --- | --- | --- | --- | --- | --- | --- | --- |
| S-001 0 h | 0 | BQL | BQL | BQL | NC | 0.0001 | BQL | BQL | NC |
| S-002 0 h | 0 | BQL |  |  |  | 0.0001 |  |  |  |
| S-003 0 h | 0 | BQL |  |  |  | 0.0001 |  |  |  |
| S-004 0.5 h | 0.5 | 1.9214 | 1.8724 | 0.4129 | 22.1 % | 1.0853 | 1.0143 | 0.0920 | 9.1 % |
| S-005 0.5 h | 0.5 | 2.2586 |  |  |  | 1.0473 |  |  |  |
| S-006 0.5 h | 0.5 | 1.4372 |  |  |  | 0.9103 |  |  |  |
| S-007 1 h | 1 | 0.9667 | 0.8543 | 0.1724 | 20.2 % | 0.5364 | 0.5301 | 0.0291 | 5.5 % |
| S-008 1 h | 1 | 0.9403 |  |  |  | 0.5555 |  |  |  |
| S-009 1 h | 1 | 0.6557 |  |  |  | 0.4983 |  |  |  |
| S-010 2 h | 2 | 0.4355 | 0.4495 | 0.0155 | 3.4 % | 0.3270 | 0.2964 | 0.0319 | 10.8 % |
| S-011 2 h | 2 | 0.4469 |  |  |  | 0.2633 |  |  |  |
| S-012 2 h | 2 | 0.4661 |  |  |  | 0.2990 |  |  |  |
| S-013 4 h | 4 | 0.2628 | 0.2996 | 0.0710 | 23.7 % | 0.1820 | 0.2276 | 0.0962 | 42.3 % |
| S-014 4 h | 4 | 0.2546 |  |  |  | 0.1627 |  |  |  |
| S-015 4 h | 4 | 0.3815 |  |  |  | 0.3382 |  |  |  |
| S-016 8 h | 8 | 0.1504 | 0.1302 | 0.0175 | 13.4 % | 0.1347 | 0.1123 | 0.0198 | 17.6 % |
| S-017 8 h | 8 | 0.1206 |  |  |  | 0.0972 |  |  |  |
| S-018 8 h | 8 | 0.1196 |  |  |  | 0.1049 |  |  |  |
| S-019 24 h | 24 | 0.0389 | 0.0369 | 0.0086 | 23.4 % | 0.0670 | 0.0565 | 0.0094 | 16.7 % |
| S-020 24 h | 24 | 0.0274 |  |  |  | 0.0536 |  |  |  |
| S-021 24 h | 24 | 0.0443 |  |  |  | 0.0488 |  |  |  |
| S-022 48 h | 48 | 0.0175 | 0.0121 | 0.0048 | 39.4 % | 0.0192 | 0.0180 | 0.0012 | 6.8 % |
| S-023 48 h | 48 | 0.0086 |  |  |  | 0.0181 |  |  |  |
| S-024 48 h | 48 | 0.0101 |  |  |  | 0.0168 |  |  |  |

BQL = Below quantitation limit (0.005 µM).

NC = Not Calculated.

Table S4. Concentrations of PCI-2022 and PCI-2050 determined in mouse tumors.

| Sample ID | Time Post Injection (hr) | Final Conc. Of PCI-2022 (nmol/g) | Mean | StDev | RSD | Final Conc. Of PCI-2050 (nmol/g) | Mean | StDev | RSD |
| --- | --- | --- | --- | --- | --- | --- | --- | --- | --- |
| S-097 0 h | 0 | BQL | BQL | BQL | NC | BQL | BQL | NC | NC |
| S-098 0 h | 0 | BQL |  |  |  | BQL |  |  |  |
| S-099 0 h | 0 | BQL |  |  |  | BQL |  |  |  |
| S-100 0.5 h | 0.5 | 0.1528 | 0.2703 | 0.1036 | 38.3 % | 0.1484 | 0.2620 | 0.0985 | 37.6 % |
| S-101 0.5 h | 0.5 | 0.3484 |  |  |  | 0.3134 |  |  |  |
| S-102 0.5 h | 0.5 | 0.3097 |  |  |  | 0.3241 |  |  |  |
| S-103 1 h | 1 | 0.1125 | 0.1892 | 0.0880 | 46.5 % | 0.0892 | 0.2246 | 0.1338 | 59.6 % |
| S-104 1 h | 1 | 0.1697 |  |  |  | 0.2279 |  |  |  |
| S-105 1 h | 1 | 0.2853 |  |  |  | 0.3567 |  |  |  |
| S-106 2 h | 2 | 0.1119 | 0.2013 | 0.0959 | 47.7 % | 0.1197 | 0.2021 | 0.1152 | 57.0 % |
| S-107 2 h | 2 | 0.3026 |  |  |  | 0.3337 |  |  |  |
| S-108 2 h | 2 | 0.1894 |  |  |  | 0.1527 |  |  |  |
| S-109 4 h | 4 | 0.2843 | 0.3049 | 0.0525 | 17.2 % | 0.3028 | 0.3081 | 0.0265 | 8.6 % |
| S-110 4 h | 4 | 0.2659 |  |  |  | 0.2847 |  |  |  |
| S-111 4 h | 4 | 0.3646 |  |  |  | 0.3368 |  |  |  |
| S-112 8 h | 8 | 0.1034 | 0.2256 | 0.1197 | 53.1 % | 0.1014 | 0.2324 | 0.1345 | 57.9 % |
| S-113 8 h | 8 | 0.3427 |  |  |  | 0.3701 |  |  |  |
| S-114 8 h | 8 | 0.2307 |  |  |  | 0.2257 |  |  |  |
| S-115 24 h | 24 | 0.1471 | 0.1812 | 0.0607 | 33.5 % | 0.1400 | 0.1975 | 0.0929 | 47.0 % |
| S-116 24 h | 24 | 0.2513 |  |  |  | 0.3047 |  |  |  |
| S-117 24 h | 24 | 0.1454 |  |  |  | 0.1478 |  |  |  |
| S-118 48 h | 48 | 0.1324 | 0.1462 | 0.0371 | 25.4 % | 0.1359 | 0.1302 | 0.0140 | 10.8 % |
| S-119 48 h | 48 | 0.1882 |  |  |  | 0.1406 |  |  |  |
| S-120 48 h | 48 | 0.1180 |  |  |  | 0.1143 |  |  |  |

BQL = Below quantitation limit (0.025 nmol/g).

NC = Not Calculated.

Table S5. Concentrations of PCI-2022 and PCI-2050 determined in mouse kidneys.

| Sample ID | Time Post Injection (hr) | Final Conc. Of PCI-2022 (nmol/g) | Mean | StDev | RSD | Final Conc. Of PCI-2050 (nmol/g) | Mean | StDev | RSD |
| --- | --- | --- | --- | --- | --- | --- | --- | --- | --- |
| S-073 0 h | 0 | BQL | BQL | BQL | NC | BQL | BQL | NC | NC |
| S-074 0 h | 0 | BQL |  |  |  | BQL |  |  |  |
| S-075 0 h | 0 | BQL |  |  |  | BQL |  |  |  |
| S-076 0.5 h | 0.5 | 36.9403 | 33.6127 | 4.9341 | 14.7 % | 63.6435 | 54.5803 | 8.5959 | 15.7 % |
| S-077 0.5 h | 0.5 | 35.9539 |  |  |  | 53.5533 |  |  |  |
| S-078 0.5 h | 0.5 | 27.9438 |  |  |  | 46.5440 |  |  |  |
| S-079 1 h | 1 | 27.8177 | 28.9934 | 4.9098 | 16.9 % | 44.1806 | 44.9121 | 8.1320 | 18.1 % |
| S-080 1 h | 1 | 24.7781 |  |  |  | 37.1706 |  |  |  |
| S-081 1 h | 1 | 34.3842 |  |  |  | 53.3851 |  |  |  |
| S-082 2 h | 2 | 25.3431 | 28.4841 | 3.3118 | 11.6 % | 39.9336 | 41.7186 | 6.6633 | 16.0 % |
| S-083 2 h | 2 | 31.9437 |  |  |  | 49.0926 |  |  |  |
| S-084 2 h | 2 | 28.1655 |  |  |  | 36.1297 |  |  |  |
| S-085 4 h | 4 | 26.3498 | 29.8176 | 3.3561 | 11.3 % | 39.4558 | 43.5304 | 3.6154 | 8.3 % |
| S-086 4 h | 4 | 30.0535 |  |  |  | 44.7808 |  |  |  |
| S-087 4 h | 4 | 33.0494 |  |  |  | 46.3547 |  |  |  |
| S-088 8 h | 8 | 23.0592 | 22.0642 | 2.5571 | 11.6 % | 29.3101 | 25.1246 | 4.4508 | 17.7 % |
| S-089 8 h | 8 | 23.9744 |  |  |  | 25.6148 |  |  |  |
| S-090 8 h | 8 | 19.1592 |  |  |  | 20.4491 |  |  |  |
| S-091 24 h | 24 | 25.6087 | 21.0358 | 3.9689 | 18.9 % | 18.9151 | 14.7594 | 3.6774 | 24.9 % |
| S-092 24 h | 24 | 19.0116 |  |  |  | 11.9261 |  |  |  |
| S-093 24 h | 24 | 18.4872 |  |  |  | 13.4371 |  |  |  |
| S-094 48 h | 48 | 18.5631 | 15.7576 | 2.7268 | 17.3 % | 6.0544 | 5.2729 | 0.7490 | 14.2 % |
| S-095 48 h | 48 | 13.1169 |  |  |  | 5.2031 |  |  |  |
| S-096 48 h | 48 | 15.5927 |  |  |  | 4.5612 |  |  |  |

BQL = Below quantitation limit (0.025 nmol/g).

NC = Not Calculated.

**Scheme 1** Synthesis of Hydrophilic Sapphyrins. Reagents and conditions: a, N,N′-disuccinimidyl carbonate/iPr_2_EtN/DCM/ rt; b, amine (RH)/DCM/ rt.

**General Procedure for the Synthesis of Sapphyrins.** All reagents and starting materials were purchased and used without further purification unless otherwise noted. Dihydroxylated sapphyrin PCI-2001 (1) and bis[2-[2-(2-methoxyethoxy)ethoxy]ethyl]amine (2) were prepared according to literature methods. ^1^H and ^13^C NMR spectra were recorded using a 300 MHz GE spectrometer with Tecmag software upgrade. UV-vis spectra were obtained on an Agilent 8453 Spectrophotometer. Column chromatography was run using ICN-Silitech 32-63 D60 Å silica gel or Sorbent Technologies Std. Activity 50-200 μm neutral alumina. Sep-Pak reverse-phase tC18 cartridge columns were purchased from Waters. The purity of all products was >90% as determined by reversed phase HPLC analysis. For biological studies, compounds were formulated by dissolution in 5% mannitol and sterile filtration. Solutions of all sapphyrins were quantified in methanol using the extinction coefficient determined for PCI-2001 (^443^ε = 598,782).

**General procedure for synthesis of PCI-2012 and PCI-2050.** In a 25 mL Schlenk tube were placed **1** (200 mg, 0.303 mmol, 1 eq), N, N’-disuccinimidyl carbonate (310 mg, 1.21 mmol, 4 eq), and a magnetic stir bar. The system was dried in vacuum at room temperature for 2 hrs. Under a stream of N_2_, anhydrous CH_2_Cl_2_ (10 mL) and diisopropylethylamine (DIEA, 313 mg, 2.42 mmol, 8 eq) were added. The reaction mixture was stirred at rt for 4 hrs. At this point, diethanolamine (191 mg, 1.82 mmol, 6 eq) or bis[2-[2-(2-methoxyethoxy)ethoxy]ethyl]amine (564 mg, 1.82 mmol, 6 eq) dissolved in 2 mL CH_2_Cl_2_ was added, and the resulting mixture was stirred for another 1 hr. The reaction mixture was concentrated to give an oily residue, which was purified by column chromatography on neutral alumina (eluent: 0.5-1.0 % MeOH in CH_2_Cl_2_) to yield a blue solid. This crude product was then dissolved in a mixture of 2 mL MeOH and 8 mL de-ionized (DI) water, and loaded onto a 60cc tC18 Sep-Pak column (Waters). After washing with 60 mL DI water, the product band was eluted with pure MeOH. Concentration of the MeOH solution gave the target sapphyrin (PCI-2012 or PCI-2050).

**Sapphyrin PCI-2012** (Yield: 62%) ^1^H NMR (300 MHz, CDCl_3_): *δ* ppm −4.90 (1H, *NH*, s), −4.60 (1H, *NH*, s), −4.28 (1H, *NH*, s), 2.13 (t, J = 7.4 Hz, 6H, CH_2_C*H*_3_), 2.25 (t, J = 7.4 Hz, 6H, CH_2_C*H*_3_), 3.00-3.12 (m, 4H, CH_2_C*H*_2_CH_2_O-), 3.40-3.55 (m, 8H, NC*H_2_*CH_2_OH), 3.60-3.70(m, 8H, NCH_2_C*H_2_*OH), 4.14 (s, 6H, C*H*_3_), 4.24 (s, 6H, *CH_3_*), 4.57 (q, J = 7.4 Hz, 4H, C*H*_2_CH_3_), 4.65-4.82 (m, 12H, C*H*_2_CH_2_*CH_2_*O- and *CH_2_*CH_3_), 11.60 (s, 2H, *aromatic*-H); 11.71 (s, 2H, *aromatic*-H); ^13^C NMR (300 MHz, CDCl_3_): *δ* ppm 12.8, 15.8, 17.8, 18.5, 21.0, 24.3, 32.9, 59.0, 63.5, 70.7, 72.0, 91.5, 98.4, 127.1, 129.7, 130.2, 132.4, 134.9, 135.4, 137.4, 138.7, 142.0, 142.8, 156.5. UV/Vis in MeOH [*λ*_max_, nm]: 446, 616, 670. FAB-MS: m/z 922.6 [M + H]^+^; FAB HRMS calcd for C_52_H_72_N_7_O_8_^+^ ([M + H]^+^): 922.5442; found 922.5480.

**Sapphyrin PCI-2050** (Yield: 61%) ^1^H NMR (300 MHz, CDCl_3_): *δ* ppm −4.90 (s, 1H, N*H*), −4.59 (s, 1H, N*H*), −4.29 (s, 1H, N*H*), 2.19 (t, J = 7.6 Hz, 6H, CH_2_C*H*_3_), 2.29 (t, J = 7.6 Hz, 6H, CH_2_C*H*_3_), 2.98-3.10 (m, 4H, CH_2_C*H*_2_CH_2_O-), 3.32 (s, 6H, OC*H_3_*), 3.39 (s, 6H, OC*H_3_*), 3.49-3.79 (48H, *polyether*, m), 4.12 (s, 6H, C*H*_3_), 4.23 (s, 6H, C*H_3_*), 4.56 (q, J = 7.6 Hz, 4H, C*H*_2_CH_3_), 4.65-4.80 (m, 12H, C*H*_2_CH_2_*CH_2_*O- and *CH_2_*CH_3_), 11.62 (s, 2H, *aromatic*-H), 11.71 (s, 2H, *aromatic*-H); ^13^C NMR (300 MHz, CDCl_3_): *δ* ppm 12.8, 15.8, 17.8, 18.5, 20.9, 24.3, 32.9, 59.0, 65.3, 70.6, 70.7, 72.0, 76.8, 77.2, 77.6, 77.7, 91.6, 98.4, 127.0, 129.8, 130.2, 132.5, 135.1, 135.5, 137.3, 138.6, 141.9, 142.7, 156.5. UV/Vis in MeOH [*λ*_max_, nm]: 445, 616, 666. ESI-MS: m/z 1330.7 [M + H]^+^; CI HRMS calcd for C_72_H_112_N_7_O_16_^+^ ([M + H]^+^): 1330.8166; found 1330.8212.

**General procedure for synthesis of PCI-2022 and PCI-2042.** The experimental procedure is similar to that for the synthesis of PCI-2012 and PCI-2050, except DMSO was used as the reaction solvent instead of CH_2_Cl_2_. The workup was carried out by first extracting the product into diethyl ether, washing with water, and concentrating the ether solution. The residue was then purified on a Sep-Pak reversed phase column to give the product.

**Sapphyrin PCI-2022** (Yield: 42%) ^1^H NMR (300 MHz, CD_3_OD): *δ* ppm 2.06 (t, J = 7.2 Hz, 6H, CH_2_C*H*_3_), 2.19 (t, J = 7.2 Hz, 6H, CH_2_C*H*_3_), 3.04 (m, 4H, CH_2_C*H*_2_CH_2_O-), 3.72 (s, 12H, C*H_2_*OH), 4.17(s, 6H, C*H_3_*), 4.36 (s, 6H, C*H_3_*), 4.57 (t, J = 6.6 Hz, 4H, C*H_2_*CH_2_CH_2_O-), 4.66-4.80 (m, 12H, *CH_2_*CH_2_CH_2_O- and *CH_2_*CH_3_), 11.11 (brs, 2H, aromatic-H), 11.36 (brs, 2H, aromatic-H). ^13^C NMR (300 MHz, CD_3_OD): *δ* ppm 12.7, 15.8, 17.7, 18.3, 21.0, 24.1, 32.8, 59.0, 62.5, 64.7, 72.0, 91.5, 98.4, 127.0, 129.6, 130.1, 132.2, 134.6, 135.2, 137.3, 138.5, 141.9, 142.7, 156.4. UV/Vis in MeOH [*λ*_max_, nm]: 449, 620, 678. FAB-MS: m/z 954.6 [M + H]^+^; FAB HRMS calcd for C_52_H_72_N_7_O_10_^+^ ([M + H]^+^): 954.5341; found 954.5373.

**Sapphyrin PCI-2042** (Yield: 76%) ^1^H NMR (300 MHz, CD_3_OD): *δ* ppm 2.06 (t, J = 7.2 Hz, 6H, CH_2_C*H*_3_), 2.19 (t, J = 7.2 Hz, 6H, CH_2_C*H*_3_), 3.04 (m, 4H, CH_2_C*H*_2_CH_2_O-), 3.27-3.39 (m, 4H, *CH_2_*OH), 3.41-3.51 (m, 2H, -C*CH*(NH)C-), 3.71-3.96 (m, 6H, -C*CH*(O)C-), 4.17(s, 6H, C*H_3_*), 4.36 (s, 6H, C*H_3_*), 4.59 (t, J = 6.6 Hz, 4H, C*H_2_*CH_2_CH_2_O-), 4.66-4.80 (m, 4H, *CH_2_*CH_2_CH_2_O-), 4.85-5.06 (m, 8H, *CH_2_*CH_3_), 5.30 (d, J = 3.0 Hz, 2H, -C*H*(OH)O-), 11.11 (brs, 2H, aromatic-H), 11.36 (brs, 2H, aromatic-H). ^13^C NMR (300 MHz, CD_3_OD): *δ* ppm 12.7, 15.8, 17.7, 18.3, 21.0, 24.1, 32.7, 58.9, 63.6, 64.0, 71.7, 72.0, 74.5, 76.7, 91.5, 97.2, 98.4, 127.0, 129.6, 130.1, 132.4, 134.8, 135.3, 137.3, 138.6, 141.9, 142.7, 156.4. UV/Vis in MeOH [*λ*_max_, nm]: 449, 621, 679. FAB-MS: m/z 1070.6 [M + H]^+^; FAB HRMS calcd for C_56_H_76_N_7_O_14_^+^ ([M + H]^+^): 1070.5450; found 1070.5440.

**Figure S11.**  **NCI Developmental Therapeutics Program tumor cell line panel data.** PCI-2050 was tested for cell inhibitory activity using the sulforhodamine B assay after two days of treatment. Mean graphs were prepared as described on the NCI website (http://dtp.nci.nih.gov/docs/cancer/cancer_data.html).


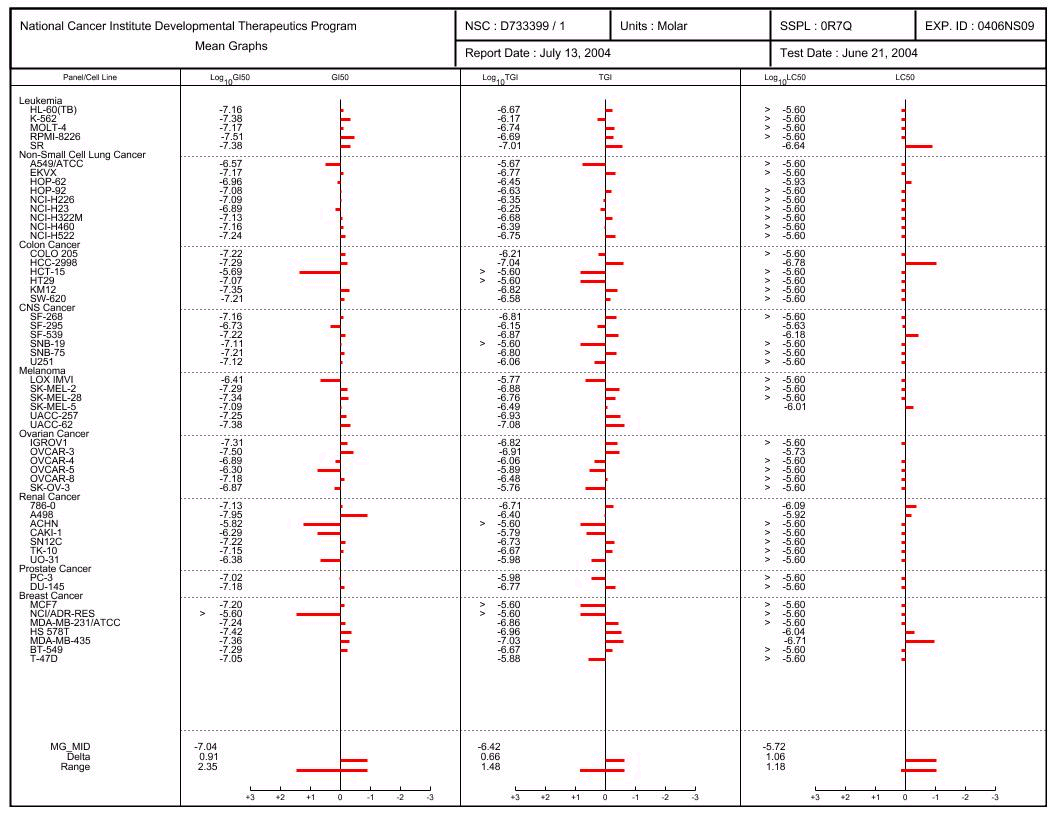


**Figure S12.**  **COMPARE Results.** The COMPARE algorithm (3) was used to identify compounds present in the NCI synthetic compound database with GI_50_ values correlating most closely to those of PCI-2050 in the cell line panel data as described (http://dtp.nci.nih.gov/docs/compare/compare.html).

Reference List

(1) Sessler J, Cyr MJ, Lynch V, McGhee E, Ibers JA. Synthetic and Structural Studies of Sapphyrin, a 22-Pi-Electron Pentapyrrolic "Expanded Porphyrin". J Am Chem Soc 1990;112:2810-3.

(2) Selve C, Ravey J-C, Stebe M-J, El Moudjahid C, Moumni EM, Delpuech J-J. Tetrahedron 1991;47:411-28.

(3) Zaharevitz DW, Holbeck SL, Bowerman C, Svetlik PA. COMPARE: a web accessible tool for investigating mechanisms of cell growth inhibition. J Mol Graph Model 2002 Jan;20(4):297-303.
